# Effect of shell structure of Ti-immobilized metal ion affinity chromatography core-shell magnetic particles for phosphopeptide enrichment

**DOI:** 10.1038/s41598-019-51995-z

**Published:** 2019-10-31

**Authors:** Anna Laura Capriotti, Michela Antonelli, Diego Antonioli, Chiara Cavaliere, Riccardo Chiarcos, Valentina Gianotti, Susy Piovesana, Katia Sparnacci, Michele Laus, Aldo Laganà

**Affiliations:** 1 Department of Chemistry, Sapienza Università di Roma, Piazzale Aldo Moro 5, 00185 Rome, Italy; 2 Department of Science and Technological Innovation, Università degli Studi del Piemonte Orientale, Alessandria, Italy; 3 INSTM, UdR Alessandria, Viale Teresa Michel 11, 15121 Alessandria, Italy; 4 CNR NANOTEC, Campus Ecotekne, University of Salento, Via Monteroni 73100 Lecce, Italy

**Keywords:** Bioanalytical chemistry, Magnetic materials

## Abstract

Magnetic materials in sample preparation for shotgun phosphoproteomics offer several advantages over conventional systems, as the enrichment can be achieved directly in solution, but they still suffer from some drawbacks, due to limited stability and selectivity, which is supposed to be affected by the hydrophilicity of the polymeric supports used for cation immobilization. The paper describes the development of an improved magnetic material with increased stability, thanks to a two-step covering of the magnetic core, for the enrichment of phosphopeptides in biological samples. Four materials were prepared featuring a polymeric shell with tunable hydrophilicity, obtained by “grafting from” polymerization of glycidyl methacrylate with 0–8.3% of polyethylene glycol methacrylate (PEGMA), the latter used to modulate the hydrophilicity of the material surface. Finally, the materials were functionalized with iminodiacetic acid for Ti^4+^ ion immobilization. The materials were analyzed for their composition by a combination of CHN elemental analysis and thermogravimetric analysis, also hyphenated to gas chromatography and mass spectrometric detection. Surface characteristics were evaluated by water contact angle measurements, scanning electron microscopy and energy dispersive X-ray spectrometry. These materials were applied to the enrichment of phosphopeptides from yeast protein digests. Peptides were identified by proteomics techniques using nano-high performance liquid chromatography coupled to mass spectrometry and bioinformatics. Qualitatively the peptides identified by the four systems were comparable, with 1606–1693 phosphopeptide identifications and a selectivity of 47–54% for all materials. The physico-chemical features of the identified peptides were also the same for the four materials. In particular, the grand average of hydropathy index values indicated that the enriched phosphopeptides were hydrophilic (ca. 90%), and only some co-enriched non-phosphorylated peptides were hydrophobic (21–28%), regardless of the material used for enrichment. Peptides had a pI ≤ 7, which indicated a well-known bias for acidic peptides binding, attributed to the interaction with the metal center itself. The results indicated that the enrichment of phosphopeptides and the co-enrichment of non-phosphorylated peptides is mainly driven by interactions with Ti^4+^ and does not depend on the amount of PEGMA chains in the polymer shell.

## Introduction

Protein phosphorylation represents a major protein post translational modification and an active switch for several biological processes, a regulatory mechanism in physiological conditions whose dysfunction has been associated to several pathological conditions^1,2^. Accordingly, the investigation of protein phosphorylation is a valuable tool to elucidate disease mechanisms and develop strategies for the early diagnosis of disease. As site-specific phosphorylation events are emerging as potential disease biomarkers^1,3^, phosphopeptides represent good candidates in biomarker discovery research^4,5^. However, the direct analysis of protein phosphorylation cannot be performed without enrichment in the sample preparation, as protein phosphorylation is a substoichiometric process and several important phosphoproteins are low abundant. Modern phosphoproteomics comprises both untargeted and targeted approaches for phosphopeptide detection, the latter emerging as promising candidates to investigate site-specific phosphorylation^6^, with both approaches relying on liquid chromatography coupled to tandem mass spectrometry (MS/MS). In either approach, enrichment of phosphorylations is a necessary process to avoid analyte suppression and is usually performed after protein digestion, on the resulting phosphopeptides^7,8^.

Although several strategies have been described for phosphopeptide enrichment, two main approaches are currently commercially exploited and extensively studied for research applications, namely metal oxide affinity chromatography (MOAC) and immobilized metal ion affinity chromatography (IMAC). While metal oxides can be directly synthetized or supported on other materials, to modulate the surface area and phosphopeptide binding capacity, in the case of IMAC materials a support is always necessary for metal ion immobilization by chelation. Regardless of the enrichment approach, the nature of the support has been extensively investigated^8^ indicating that the support architecture is not completely inert, as hydrophilic supports can improve the phosphopeptide enrichment selectivity by reducing the hydrophobic interactions which are responsible of unspecific binding of non-phosphorylated peptides^9^. Support hydrophilicity has been suggested to improve phosphopeptide enrichment selectivity for IMAC^4,10–18^, but also for some MOAC materials^19–22^, metal-organic framework (MOF) materials^23–25^ and hybrid MOAC-IMAC materials as well^26^. This observation has prompted the development of hydrophilic materials for phosphopeptide enrichment; for instance, polydopamine was extensively used to prepare MOF^23–25^, IMAC supports^10–14,27–30^, MOAC supports^31^ or frameworks further derivatized with linkers, such as adenosine triphosphate^32^. Although some studies were performed by high performance liquid chromatography (HPLC) coupled to mass spectrometry (MS)^11,12,17,23,33^ most works in this field analyze samples directly by Matrix-Assisted Laser Desorption/Ionization without any chromatographic separation^10,13,14,16,21,22,24,25,31,32,34,35^, which does not allow a comprehensive characterization of a complex tryptic digest.

However, despite the drive for developing new hydrophilic materials, still a systematic evaluation of phosphopeptide enrichment selectivity was never comparatively evaluated as a function of the hydrophilicity of the material. Therefore, in this work the phosphopeptide enrichment selectivity was systematically evaluated on a complex tryptic digest, to assess if a change in the hydrophilicity of an IMAC material would impact the phosphopeptide enrichment selectivity and to what extent. A multishell magnetic functional material was developed, starting from a promising one previously employed for phosphopeptide enrichment^33^, and the preparation was modified to allow modulation of hydrophilic moieties displayed on the surface of the material. This resulted in four IMAC materials, with increasing hydrophilicity. Among the possible metal cations suitable for IMAC enrichment of phosphopeptides, Ti^4+^ was chosen, as it was recently demonstrated to perform better than other cations commonly employed in IMAC phosphopeptide enrichment^36^.

## Results and Discussion

**Figure 1:**
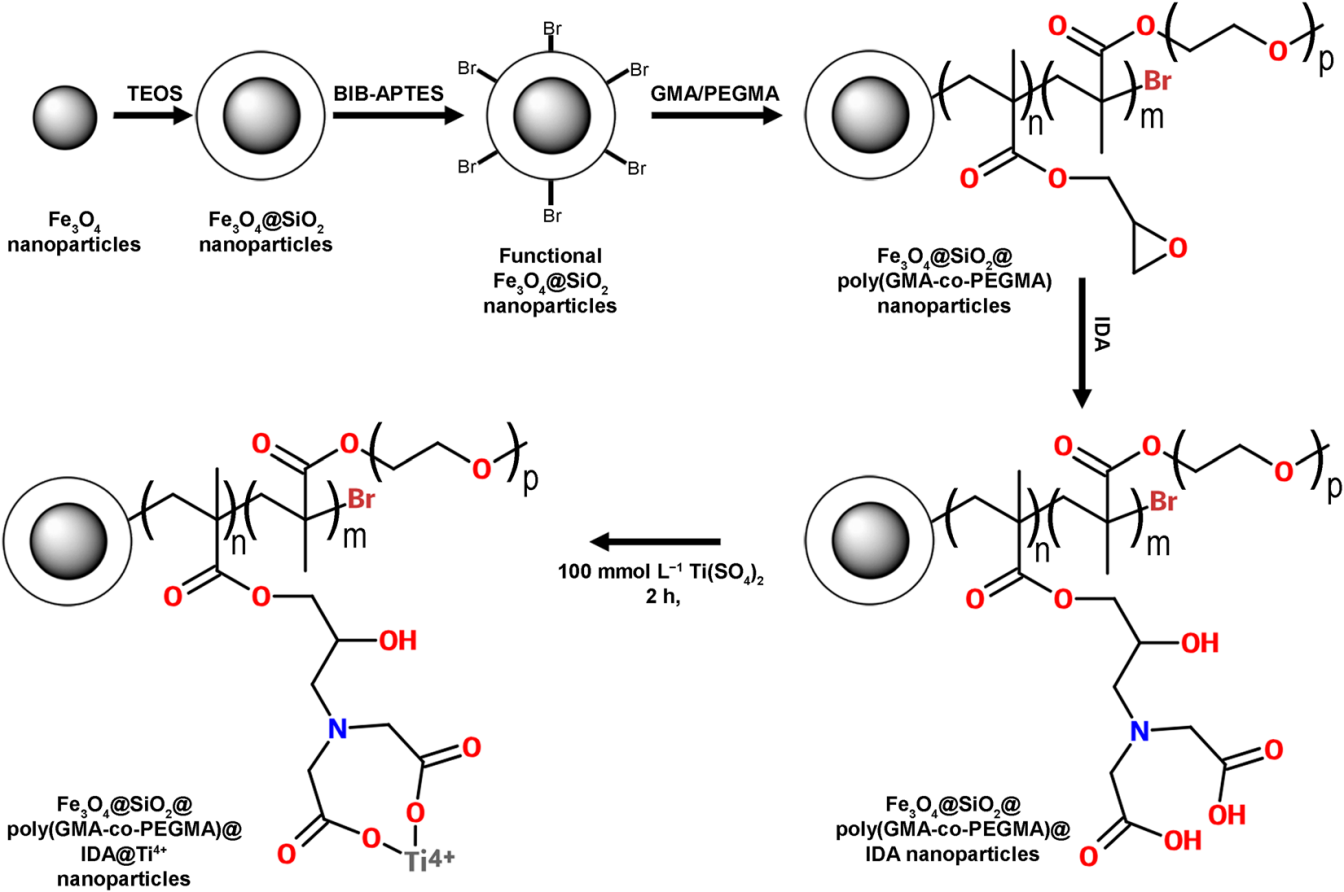
Representation of the synthetic route for preparation of magnetic polymeric nanoparticles. The hydrophilicity of the surface material was modulated by changing the ratio between GMA and PEGMA.

Core-shell magnetic materials were prepared starting from magnetite nanoparticles as described in detail in Fig. 1. The reaction sequence is similar to the one previously reported^33^, although a better procedure was adopted in the coverage of magnetite nanoparticles with bromoisobutyryl-3-aminopropyltriethoxysilane (BIB-APTES). Such precaution was introduced to improve the structure of the magnetic nanoparticles and increase the long-term stability of the materials, as it was previously observed that the magnetic properties of magnetic materials drastically reduced after several months from preparation, with a subsequent decrease in peptide binding^10,12,33^. A loss of magnetization and phosphopeptide enrichment performance is a common issue, and it was attributed not only to the formation of several layers around the magnetic core, but most probably to a chemical reaction of Ti^4+^ with the Fe_3_O_4_ magnetic core, as Ti^4+^ is loaded under very harsh acidic conditions and stored in acidic medium. Accordingly, it appears that the full coverage of the Fe_3_O_4_ magnetic core is of outmost importance to improve the long term stability of these systems^10^. Therefore, in the present case, the coverage of the magnetic core was performed in two steps, as described in the experimental part, thus assuring a more homogeneous coverage of the magnetite nanoparticle surface.

To further modulate the performances of the shell material with respect to the interaction and selectivity to phosphopeptides, the present work focuses on four magnetic core-shell materials, which varied in the shell structure. More specifically, two different monomers were employed in the “grafting from” reactions, namely the polyethylene glycol methacrylate (PEGMA), with an average molar mass of 1100 g/mol, and glycidyl methacrylate (GMA), thus leading to four materials, marked M0-M3, where the number identifies the PEGMA percentage that ranges from 0% (M0) to 8.3% (M3). The change in the percent of PEGMA in the shell allowed to modulate the hydrophilicity of the outer shell of the nanoparticles by changing the amount of polyethyleneoxyde (PEG) containing monomeric units aside from the GMA units.

Then iminodiacetic acid (IDA) was reacted with the dangling epoxy groups and Ti^4+^ ions were bound to the coordination sites thus leading to the final Fe_3_O_4_@silica@poly(GMA-co-PEGMA)@IDA@Ti^4+^.

### Multishell magnetic material characterization

As these materials are magnetic in nature and any pretreatment would modify their properties, few techniques are suitable for the material characterization. The materials were analyzed for their composition by a combination of CHN elemental analysis and thermogravimetric techniques involving both thermogravimetric analysis (TGA) and TGA hyphenated to gas chromatography (GC) and MS detection. Surface characteristics were estimated by water contact angle measurements.

The water contact angle data (Table 1) for the final samples M0-M3 indicated a highly hydrophilic character. However, due to the high limit of quantification, a differentiation along the series could not be done. Accordingly, to obtain indirect information regarding the chemical composition of the shell material of samples M0-M3, the water contact angle determination was performed on the precursor samples EM0-EM3. For the latter samples, the contact angle decreases as the PEGMA amount increases in agreement with an increasing hydrophilic character promoted by the incorporation of PEGMA units along the polymer chains.

**Table 1:** Characterization data of samples M0-M3 and EM0-EM3.

	C (% w/w)	H (% w/w)	N (% w/w)	Water contact angle (°)	IDA Opening Yield (%)	PEGMA (% w/w)	PEGMA (% mol)
M0	48.36	6.88	2.36	<10	24 ± 6	—	—
M1	46.56	6.89	2.37	<10	40 ± 6	1.2 ± 0.7	0.3
M2	46.44	6.90	2.50	<10	35 ± 5	3.1 ± 0.6	0.6
M3	43.41	6.24	2.06	<10	32 ± 6	4.1 ± 0.9	0.8
EM0				78 ± 3	—		
EM1				73 ± 2	—		
EM2				70 ± 2	—		
EM3				67 ± 4	—		

TGA and TGA hyphenated to GC-MS allowed obtaining information about the IDA functionalization percentage, which is a critical parameter affecting the efficiency of the material in phosphopeptide enrichment.

**Figure 2:**
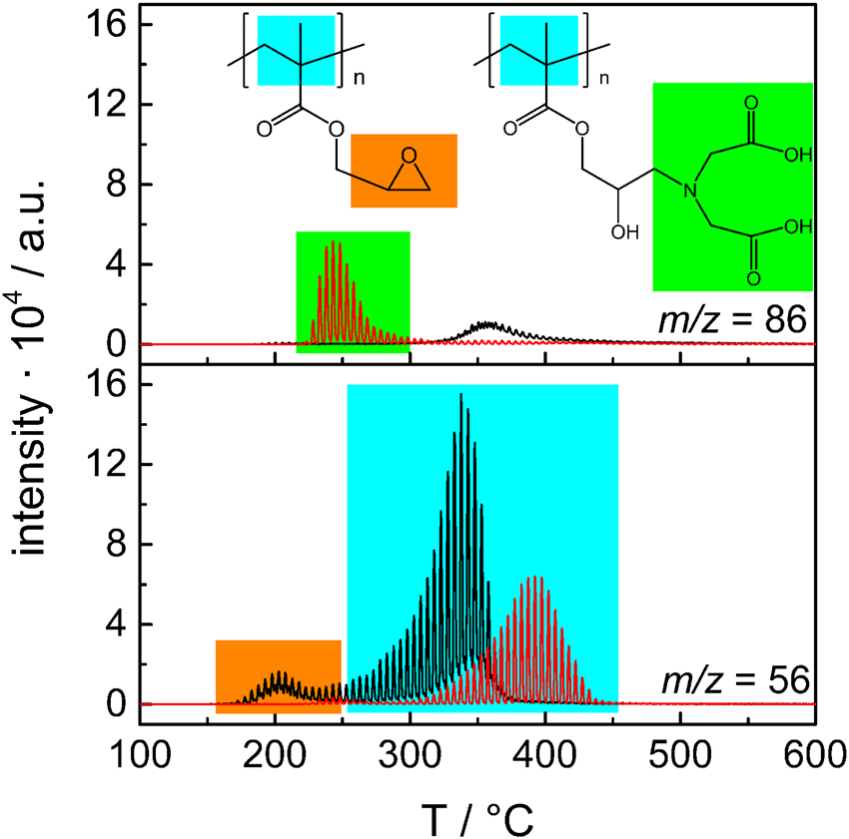
TGA-GC-MS chromatograms of the materials before (samples EM1, black curves) and after (samples M1, red curves) the epoxide ring opening reaction with IDA.

Preliminary analyses were carried out to obtain TGA-GC-MS setup optimization and synchronization^37^ and to identify fragments suitable to characterize the polymeric part of the multi-shell magnetic materials EM0-EM3 and M0-M3. The TGA-GC-MS analysis was carried out by heating these materials at 20.0 °C min^−1^ from room temperature to 600 °C, setting the mass spectrometric acquisition in full scan mode. Figure 2 reports the TGA-GC-MS chromatograms of samples EM1 and M1, as typical examples, for two significant *m/z* signals extracted from the full-scan data chromatograms. The fragment at 86 *m/z* was assigned to the fragmentation of polymeric units containing the IDA moiety and was present in samples M1 (Fig. 2, red curves). Obviously, this fragment was absent in the chromatograms of EM1 sample. The fragment at 56 *m/z* showed a first peak that evolved with the maximum at 200 °C and was present only in the EM1 chromatogram (Fig. 2, black curve). It was assigned to a fragment containing the epoxide group (Fig. 2, orange marks). Furthermore, intense peaks were observed at about 380 °C in the chromatograms of both EM1 and M1 samples. This peak derived from the fragmentation of the polymeric PEGMA chains.

Although the diagnostic peaks at 56 and 86 *m*/*z* provided information about the epoxy ring opening and IDA functionalization, a fragment characteristic for the PEGMA units was not disclosed.

**Figure 3:**
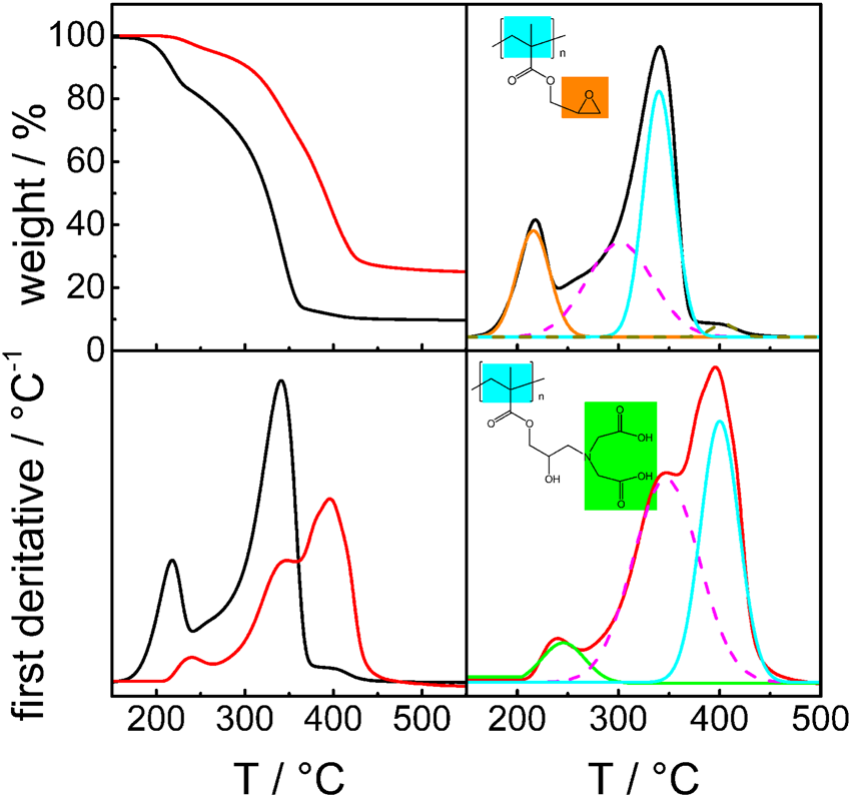
TGA curves (left side, upper part) and first derivative (left side, bottom part) of samples EM0 (black curve) and M0 (red curve). Deconvolution peaks (right side) of samples EM0 (black curve) and M0 (red curve).

The percentage of epoxy groups reacted with IDA could not be directly estimated from the TGA-GC-MS analysis due to lack of standard materials with known amounts of IDA. However, an estimation of this percentage was obtained by TGA after identification from TGA-GC-MS chromatograms of the start and end temperatures of the peak of the epoxy groups before reaction with IDA and the peak relevant to the loss of IDA after the epoxy ring opening reaction. These data can be extracted from the TGA curves by deconvolution of the first derivative peaks as shown in Fig. 3 for samples EM0 and M0. The percentage of epoxy groups reacted with IDA was given by the ratio between the areas of the peaks and are collected in Table 1. The relatively low percentage of epoxy groups reacted with IDA, that ranges between 20 and 35%, was in qualitative agreement with the literature^38^ taking into account the competition of water with IDA once the reaction is performed in a basic environment.

Finally, the percentages of PEGMA were estimated from CHN elemental analysis (Table 1). The weight percentage of PEGMA that co-polymerized with the GMA increased from 1.2 to 4.1 as the amount of PEGMA in the reaction mixture increases.

**Figure 4:**
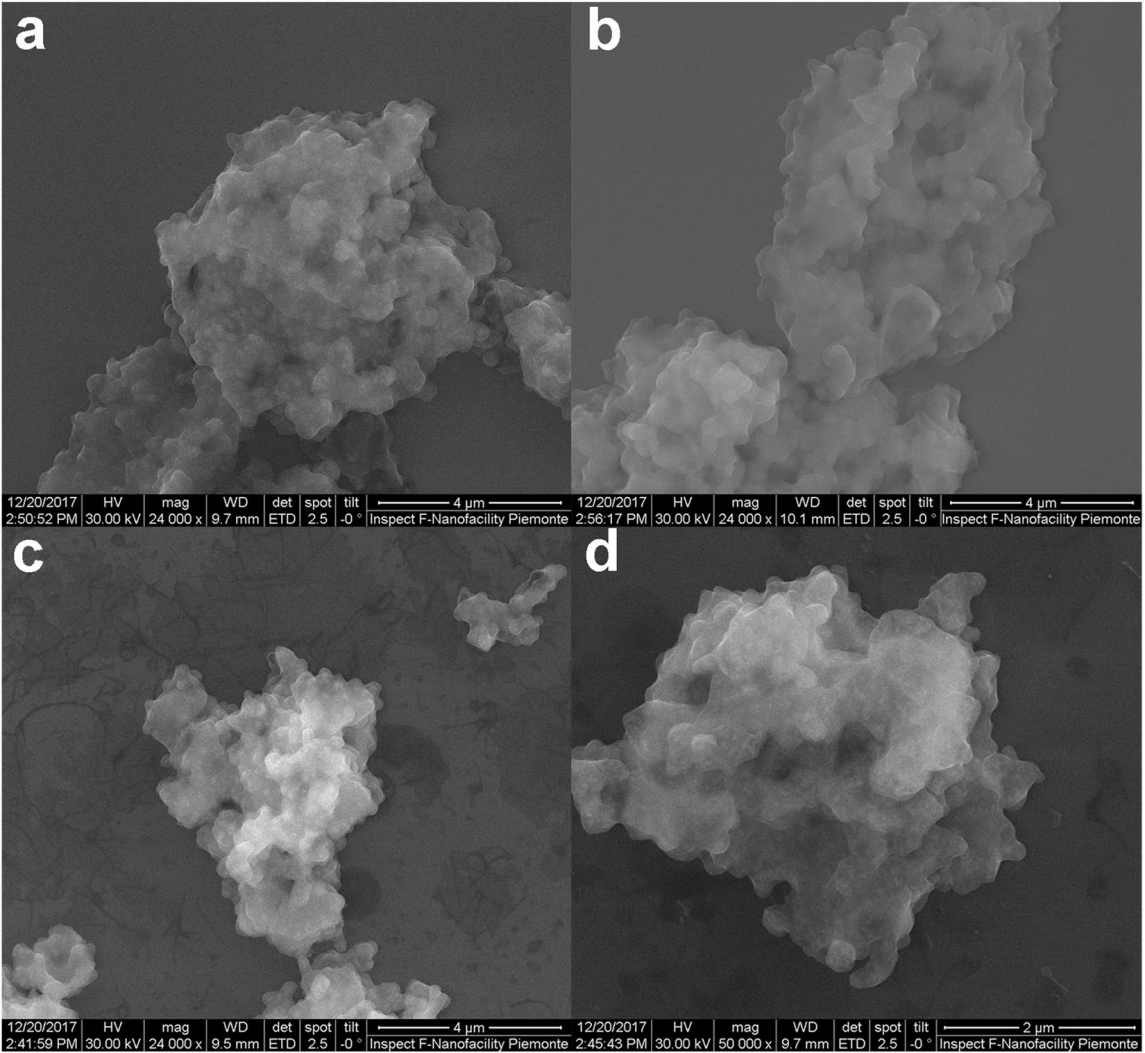
SEM images of samples M0-M3.

Scanning electron microscopy (SEM) analysis showed that the amount of polymer in the four materials increased from M0 to M3 (Fig. 4 and Fig. S1–S4). Additionally, the images showed that in the final material, the polymeric material fully covered the aggregates, which were approximately 100 nm large (Fig. S5). The aggregates, in turn, consisted of smaller particles, the original magnetic nanoparticles, which were 15 nm large (Fig. S6).

As a final consideration, a microanalysis was performed on the prepared materials by Energy Dispersive X-ray Spectrometry (EDS). Similar amounts of Ti on the surface were measured for the four materials, i.e; 1.9% ± 0.1% for M0, 2.5% ± 0.2% for M1, 2.3% ± 0.1% for M2 and 1.7% ± 0.1% for M3. Consequently, the four materials had similar amounts of Ti, which was responsible of the selective interaction with the phosphopeptides during enrichment. Such result agreed with the preparation plan, as the amount of chelating agent (IDA) was the same in all the preparations to avoid effects related to differences in the chelation site density. EDS examination also showed the homogeneous distribution of Ti on the four materials.

### Study on the effect of the shell composition on the enrichment selectivity of Ti^4 +^-IMAC magnetic materials

The four magnetic materials were employed for enrichment of phosphopeptides and their performance was related to the different hydrophilicity of the IMAC support. A complex sample, which could mimic the sample complexity of a real-world shotgun proteomics experiment, was employed to better highlight any potential difference in performance. A typical mono-dimensional shotgun proteomics approach was chosen for sample analysis, using an optimized bead-to-sample ratio previously developed for a similar material. In fact, it is known that the selectivity has a Gaussian-like distribution, with poor enrichment performance when too much or too less beads are used for enrichment^39^. In this way, known differences in selectivity due to recovery capacity of the material could be avoided, while the normalized amount of Ti on the four materials eliminated possible differences in the phosphopeptide enrichment due to different amount of Ti on the material. The results of peptide identifications are reported in Table 2 and Table S1. What is apparent from the data is that qualitatively the peptides identified by the four systems were comparable, both from the point of view of total peptide identifications and the total phosphopeptide identifications. All systems provided between 1606–1693 phosphopeptide identifications, regardless of the nature of the IMAC support. Similarly, the total number of peptide identifications was in the range 3005–3475, again with no significant difference related to the material. Furthermore, the selectivity (which was calculated as the ratio between the phosphopeptides and the total peptide identifications, in percentage) did not appear to be significantly affected by the change in material hydrophobicity. For all systems, the selectivity was within 47–54%. A deeper mining into the distribution of the phosphopeptides across the results by the four materials further supported that the average performance is not affected by the increased hydrophilicity of the surface of the IMAC material. In fact, more than 53% of the identified phosphopeptides are common to all experiments, and the individual contribution of materials M0-M3 ranges between 1.6–7.2% (Fig. S7a). A similar trend was observed for the co-enriched peptides, for which more than 56% were common to all the experimental conditions (Fig. S7b).

**Table 2:** Phosphopeptide and peptide identifications in the four materials with increasing hydrophilicity (M0-M3).

Material	Experimental replicate	Phosphopeptides identifications	Total peptides identifications	Selectivity %
M0	A	1612	3005	54
	B	1692	3134	54
	C	1688	3312	51
M1	A	1653	3201	52
	B	1606	3198	50
	C	1623	3475	47
M2	A	1693	3267	52
	B	1666	3431	49
	C	1638	3051	54
M3	A	1633	3081	53
	B	1683	3288	51
	C	1659	3381	49

**Figure 5:**
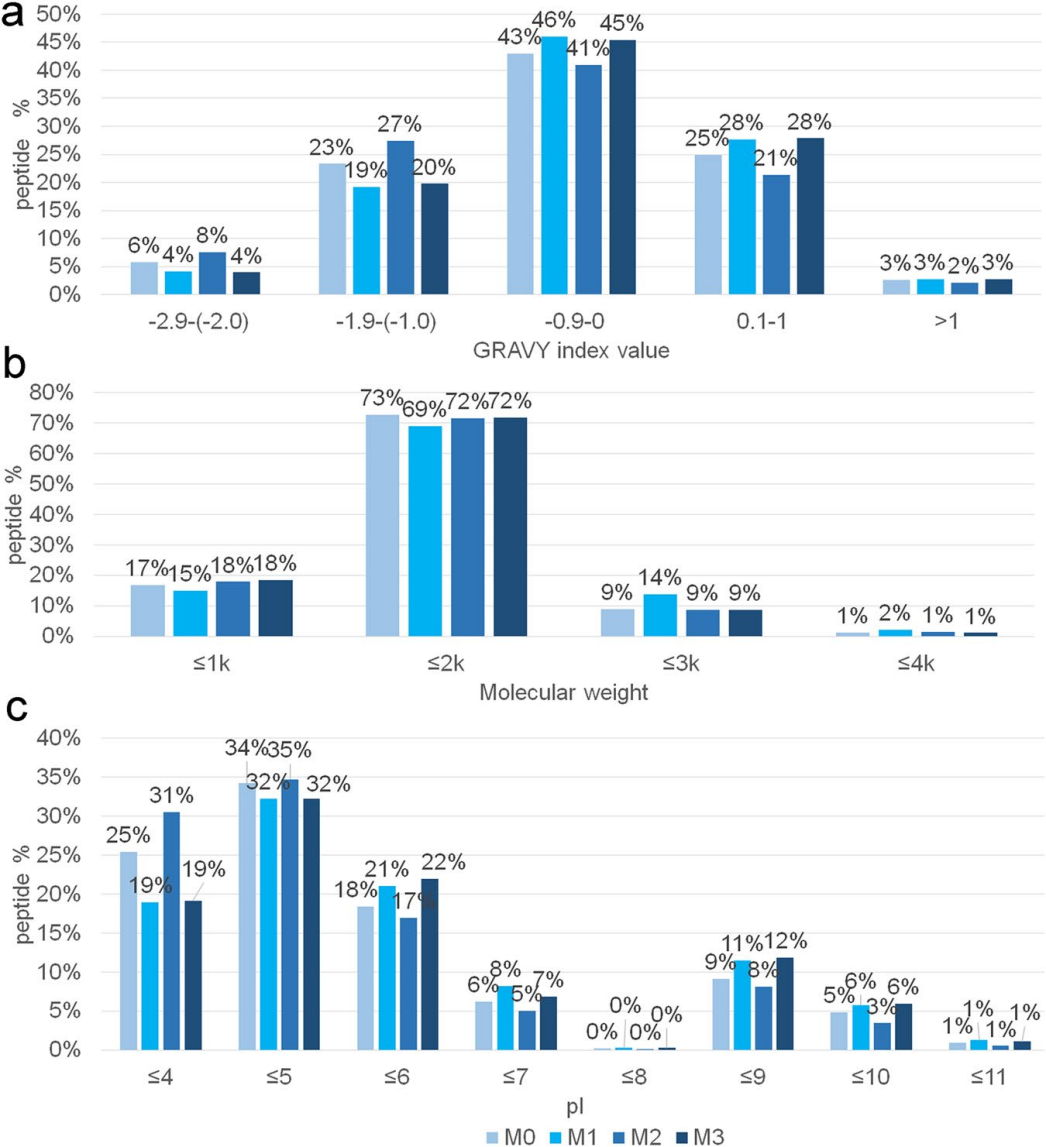
Distribution of the physico-chemical features of the co-enriched peptides (non-phosphorylated peptides) by the four material (M0-M3) with increasing hydrophilicity: (**a**) GRAVY index values; (**b**) molecular weight; (**c**) Pi.

As no significant difference was detected in either the total number of identified phosphopeptides or in the selectivity of the process, the result from the shotgun phosphoproteomics analysis was that no clear effect could be attributed to the hydrophilicity of the IMAC support. Consequently, the efficiency of phosphopeptide enrichment by a Ti^4+^-IMAC system appeared to driven by the complexation centers themselves rather than by the surface on which they were anchored to. The major role played by the metal coordination center rather than the polymeric surface might be obvious for the specific interaction with the phosphopeptides, nevertheless it appeared to govern the co-enrichment of non-phosphorylated peptides as well. This was apparent not only from the calculated selectivity for the four materials (Table 2) but also from a deeper evaluation of the physico-chemical features of the identified peptides. In this sense, the grand average of hydropathy (GRAVY) index value was calculated for all the identified peptides, together with the molecular weight and the isoelectric point (pI). Such investigation, again, indicated no clear effect could be attributed to the different hydrophilicity of the polymer surfaces. For the phosphopeptides, the GRAVY value analysis was reported in Fig. S8a and showed no remarkable difference between the four tested materials. In fact, the enriched phosphopeptides were hydrophilic, mainly with values between 0 and −1.9 (85–86%) or very hydrophilic (7–9%). The same analysis performed on the co-enriched peptides also showed no significant different behavior between the four materials (Fig. 5a). In this case, the distribution of GRAVY values was shifted also including some hydrophobic peptides with GRAVY values between 0.1–1 (21–28%) and still there was a large component of hydrophilic peptides with GRAVY values in the range −1.9–0 (65–68%) and some very hydrophilic peptides (4–8%). Despite the increase in hydrophilicity of the polymer support, the co-enrichment of hydrophobic peptides could still be observed and no peculiar difference was manifest between materials M0-M3. This clearly indicated that the co-enrichment phenomenon was not due to interaction with the polymer but it could be more likely attributed to a direct interaction with the Ti^4+^ cation.

To further shed light on the co-enrichment process, the distribution of pI was considered. Such evaluation is also significant, as another important cause of unspecific enrichment has been attributed to peptides acidity and possibility of coordination with the metal of IMAC systems^40^. The phenomenon of a co-enrichment driven by unspecific coordination to the cation was confirmed as well in this systematic study, as both phosphopeptides and co-enriched non-phosphorylated peptides had a pI ≤ 7, i.e. 76–80% for phosphopeptides (Fig. S8c), 80–88% for the co-enriched peptides (Fig. 5c). Thus, the distribution was very similar between phosphopeptides and co-enriched peptides, and a bias for acidic peptide binding was shown for the non-phosphorylated co-enriched peptides as well. Therefore, the enrichment system displayed a well-known bias for acidic peptides binding, with a trend which was slightly larger for the co-enriched peptides. Such effect could account for the non-selective binding of the Ti^4+^-IMAC systems, indicating the co-enrichment of non-phosphorylated peptides was not driven by interaction with the polymeric support, i.e. due to hydrophobic interaction, but it could be attributed to the interaction with the metal center itself.

Finally, the molecular weight distribution did not show differences between phosphopeptides (Fig. S8b) and non-phosphorylated peptides (Fig. 5b).

In conclusion, in this work four materials with increasing hydrophilicity were prepared to elucidate the role of the polymeric support material in Ti^4+^-IMAC phosphopeptide enrichment. Despite the several reports indicating an improved performance and better selectivity for hydrophilic supports, the systematic study described in this paper showed that no clear effect on either the total number of identified phosphopeptides or on the enrichment selectivity could be attributed to the varying hydrophilicity of the support. The four materials behaved in the same manner, thus showing that the unspecific binding of non-phosphorylated peptides is a phenomenon which is not driven by hydrophobic interactions with the polymer support but with the metal center itself. An investigation of the physico-chemical properties of the identified peptides did not show significant differences between the materials either, and no increased co-enrichment of hydrophobic peptides was observed as a function of the decreased hydrophilicity of the material. A closer look on the pI of peptides indicated a bias for acidic peptide binding, a phenomenon which was previously described and that could be accounted for the unspecific interaction with non-phosphorylated peptides.

## Material and Methods

### Materials and chemicals

All chemicals, reagents and organic solvents of the highest grade available were purchased from Sigma-Aldrich (St. Louis, MO, USA) unless otherwise stated. The functional triethoxysilane BIB-APTES, bearing an ATRP initiating site, was obtained from a condensation reaction between 3-aminopropyltriethoxysilane (APTES) and 2-bromoisobutyrylbromide (BIBB) as previously reported^41^. Poly(ethylene glycol) methacrylate (PEGMA) with a molar mass of 1100 g/mol and glycidyl methacrylate (GMA) were purchased from Sigma-Aldrich. Trypsin/Lys-C Mix Mass Spec Grade was provided by Promega (Madison, WI, USA). MilliQ water was prepared by arium 611 VF system from Sartorius (Göttingen, Germany).

### Synthesis and characterization of Fe3O4@silica@poly(GMA-co-PEGMA)@IDA@Ti^4+^ nanoparticles

Magnetite nanoparticles were prepared based on the procedure reported in Capriotti *et al*. work^33^ performing some modification in the treatment times to obtain a stability improvement. In details a co-precipitation reaction of ferrous and ferric ion solutions in a basic aqueous solution was performed. A solution with a molar ratio of ferrous and ferric ion 1:2. was prepared and added drop wise into a deoxygenated aqueous NH_3_ solution (pH = 7) under stirring with the temperature fixed at 50 °C. The alkaline solution was heated at 80 °C and stirred under nitrogen flow for 2.0 h, then the magnetic nanoparticles were precipitated with the help of a magnet and the supernatant was removed by decantation. The particles were washed five times with purified deoxygenated dH_2_O and then re-dispersed in 200 mL deoxygenated dH_2_O.

The Fe_3_O_4_ particles were then covered by a silica shell^33,42–44^. Two-hundred mg of magnetic nanoparticles were dispersed in a solution obtained by diluting 300 mL of dH_2_O with 9.0 mL of 25%aqueous NH_3_ solution and 651.0 mL of ethanol.

The obtained Fe_3_O_4_@silica nanoparticles were functionalized, using triethoxysilane BIB-APTES, to expose at the surface bromine containing groups. This dispersion was homogenized by ultrasonic application in a water bath and then introduced in a 500.0 mL round-bottom flask equipped with reflux condenser, thermometer and mechanical stirrer. The dispersion was heated to 30 °C under nitrogen flow and 3.0 mL tetraethoxysilane (TEOS) were slowly added to the reaction mixture and the system was let to react under constant stirring. After three hours, another aliquot of 3.0 mL of TEOS was added and the mixture reacted for 3.0 hours. The silica precursor is added in two steps so as to avoid the formation of Stöber nanoparticles without magnetic core. Finally, 2.0 mL of triethoxysilane BIB-APTES were added and the reaction was allowed to proceed for 16 h. At the end of the reaction, the magnetic nanoparticles were precipitated with the help of a magnet, the supernatant was removed by decantation and the particles were re-dispersed in absolute ethanol. This procedure was repeated five times to remove all unreacted reagents. Finally, the particles were redispersed in 40.0 mL absolute ethanol.

A copolymerization by surface initiated activator regenerated by electron transfer–atom transfer radical polymerization (ARGET-ATRP)^45^ of GMA and PEGMA monomers was employed to obtain polymer shells on the surface of the functional Fe_3_O_4_@silica nanoparticles (Fig. 1). By modulation of the ratio between GMA and PEGMA four materials were prepared. In details, EM0, containing 0.0 mol% PEGMA, EM1, with 1.5 mol% PEGMA, EM2, with 3.3 mol% PEGMA, and EM3, with 8.3 mol% PEGMA.

The last step was the functionalization of the nanoparticles with IDA. The active functional group able to chelate the Ti^4+^ was obtained by the opening reaction of the epoxide rings^33^ with the IDA to obtain the M0-M3 samples.

All samples were characterized by SEM (Inspect F SEM-Field Emission Gun, FEI), with a beam diameter of 3 nm.

### TGA-GC–MS analysis

Materials were placed in open alumina crucibles. The thermogravimetric analysis (TGA) run was performed by heating the sample at 20 °C min^−1^. He at flow rate 50.0 mL min^−1^ was used as carrier gas. Gas chromatography (GC)–MS separation was obtained by a Finnigan Trace GC-ULTRA and Trace DSQ. The column was a Phenomenex DB5–5ms (30 m, 0.25 mm i.d., 0.25 μm thickness), injector was in splitless mode and the temperature was 250 °C. He as carrier gas flowed at 1.0 mL min^−1^. The MS transfer line was set at 280 °C and the oven temperatures at 150 °C, respectively.

The transfer lines from the TGA to the interface and from the interface to the GC were set at 200 °C. The temperature of the interface was150 °C, and the sampling frequency was 30 s^−1^. The sampled gas from the loop to the waste was switched after 10 s and the capacity of the injection loop was 2.5 mL. The MS signal was acquired in positive electron ionization mode with ionization energy of 70.0 eV and the ion source temperature of 250 °C. In scouting experiments, the acquisition was performed in Full-Scan mode in the 50–350 *m*/*z* range. In the final method, the acquisition was also performed in Selected Ion Monitoring (SIM) mode acquiring signals at 86 *m*/*z* (IDA signal) and 56 *m*/*z* (polymeric chain signal).

### Elemental analysis: C, H, N

EA3000/DF (EuroVector, Milan, Italy) in CHN configuration was used for the elemental analyses. Reaction tube and GC oven temperatures were 980 and at 100 °C, respectively. The helium flow was 80 ml min^−1^. Oxygen (12 mL) was injected at 35 kPa. The run time was 400 s and the retention time of the gases were 33.0 s for N_2_, 52.0 s for CO_2_, 170.0 s for H_2_O. Atropine sulfate was the standard for the instrument calibration. Samples (0.5–1.5 mg) were put into a tin capsule (3.5 × 5 mm) closed leaving out the air and analyzed.

### Contact angle measurements

Water contact angle measurements were performed using an optical tensiometer Attension Theta.

### Yeast protein extraction, digestion and phosphopeptide enrichment

Yeast from *Saccharomyces cerevisiae* Type I was purchased from Sigma-Aldrich, adapting the procedure previously described^46^. One g powder was rehydrated overnight with 1 mL water at 30 °C. After centrifugation at 8100 × g for 100 min, 200 mg of the resulting yeast pellet was lysed with 300 mg acid washed glass beads (425–600 µm, Sigma-Aldrich) using 400 µL of cold lysis buffer (8 mol L^−1^ urea in 50 mmol L^−1^ Tris-HCl, pH 8, added with 1 tablet cOmplete™, Mini, EDTA-free Protease Inhibitor Cocktail (Sigma-Aldrich) and 1 tablet PhosSTOP phosphatase inhibitor cocktail (Sigma-Aldrich), used according to the manufacturer’s instructions). The sample was subjected to 10 cycles of 1 min vortex and 1 min on ice to break the yeast cells, then it was centrifuged at 20,000 × g at 4 °C for 15 min and the clear supernatant transferred into a new tube. Protein concentration was determined by the Bradford assay. Digestion was performed as previously described^46^ on 1 mg protein samples using a two-step digestion by Lys-C and trypsin.

Before use, 5 mg magnetic polymeric nanoparticles were twice conditioned with 200 µL of loading buffer (acetonitrile/H_2_O 50:50 (*v*/*v*), 0.1% trifluoroacetic acid (TFA) under slight agitation (Digital Vortex-Genie 2 by Scientific Industries, Bohemia, NY, USA) for 2 min. Then, 1 mg yeast digest was reconstituted in 300 µL of the loading buffer and incubated with 5 mg of magnetic polymeric nanoparticles for 30 min under slight agitation. After three washing operations with 200 µL of the loading buffer (2 min shaking), bound phosphopeptides were eluted twice with 200 µL of 1.5% NH_3_ (aq) under shaking for 5 min. To retrieve the magnetic materials, sample were briefly centrifuged for 30 s (14000 × g) then a permant magnet (Nd-Fe- B, 25 mm × 5 mm, by Supermagnete, Gottmadingen, Germany) was applied close to the vial. Combined eluates were acidified with 2.5% TFA to pH 2.5, desalted on C18 cartridges (Bond Elut C18, Agilent), dried down in a Speed-Vac SC250 Express (Thermo Savant, Holbrook, NY, USA) and dissolved with 100 µL 0.1% formic acid (FA) before injection. All samples were prepared in triplicate.

### NanoHPLC-MS/MS analysis and peptide identification

Twenty µL of each sample were separated by reversed phase chromatography by a Dionex Ultimate 3000 (Dionex Corporation Sunnyvale, CA, USA). Samples were preconcentrated on a C18 trapping column (Acclaim® PepMap100) loading with premixed mobile phase H_2_O/acetonitrile 98:2 (*v*/*v*) containing 0.1% (*v*/*v*) TFA at a flow-rate of 10 μL min^−1^. After loading, the sample was separated on a 25 cm long fused silica nanocolumn (25 cm × 75 μm id) packed with Acclaim-C18 particles (2.2 μm particle size) and an outlet organic monolithic frit^47^. The LC system was operated at 250 nL min^−1^ and at 25 °C. The employed mobile phases for peptide separation were H_2_O with 0.1% FA (phase A) and acetonitrile with 0.1% FA (phase B). The following linear gradient was employed for peptide separation: 2% B (0–5 min), 5% B (5–7 min), 35% B (7–97 min), 80% B (97–100 min), followed by 20 min washing at 80% B and 45 min equilibration at 2% B.

The chromatograph was directly interfaced by nanoelectrospray ionization to a LTQ-Orbitrap XL mass spectrometer (Thermo Scientific, Bremen, Germany) in the *m*/*z* range of 400–1800 Da and 60,000 (Full Width Half Maximum at *m*/*z* 400) resolution for the full scan. Spectra were acquired in top 5 data dependent mode rejecting + 1 and unassigned charge states, and fragmenting precursor ions by collision-induced dissociation at normalized collision energy of 35%, and an isolation window of 2 *m*/*z*. To minimize redundant spectral acquisitions, dynamic exclusion was enabled with a repeat count of 1 and a repeat duration of 30 s with exclusion duration of 60 s. For each sample, three technical replicates were performed.

The acquired raw MS/MS data files from Xcalibur software (version 2.2 SP1.48, Thermo Fisher Scientific) were searched against Uniprot database by Proteome Discoverer software (version 1.3, Thermo Scientific) and the Mascot (v.2.3.2, Matrix Science) search engine, as previously described^12^, using SwissProt and the S. cerevisae taxonomy (7904 entries).

## Supplementary information

Supplementary information accompanies this paper and includes SEM images of the M0-M3 materials, list of identified peptides in each technical replicate, graphs of the physicochemical features of the identified phosphopeptides

Table S1
