# Supplementary material for: Effect of shell structure of Ti-immobilized metal ion affinity chromatography core-shell magnetic particles for phosphopeptide enrichment
Source: Sci Rep. 2019 Oct 31;9:15782. doi: 10.1038/s41598-019-51995-z (PMC6823385; doi:10.1038/s41598-019-51995-z)
Supplement: Supplementary file 1 — Supplementary information accompanies this paper and includes SEM images of the M0-M3 materials, list of identified peptides in each technical replicate, graphs of the physicochemical features of the identified phosphopeptides [file 41598_2019_51995_MOESM1_ESM.pdf]

Effect of shell structure of Ti-immobilized metal ion affinity chromatography core-shell magnetic particles for phosphopeptide enrichment

Anna Laura Capriotti[1], Michela Antonelli[1], Diego Antonioli[2,3], Chiara Cavaliere[1], Riccardo Chiarcos[2], Valentina Gianotti[2,3], Susy Piovesana*[1], Katia Sparnacci[2,3], Michele Laus[2,3], Aldo Laganà[1]

[1] Department of Chemistry, Sapienza Università di Roma, Piazzale Aldo Moro 5, 00185 Rome, Italy

[2] Department of Science and Technological Innovation, Università degli Studi del Piemonte Orientale

[3] INSTM, UdR Alessandria, Viale Teresa Michel 11, 15121 Alessandria, Italy.

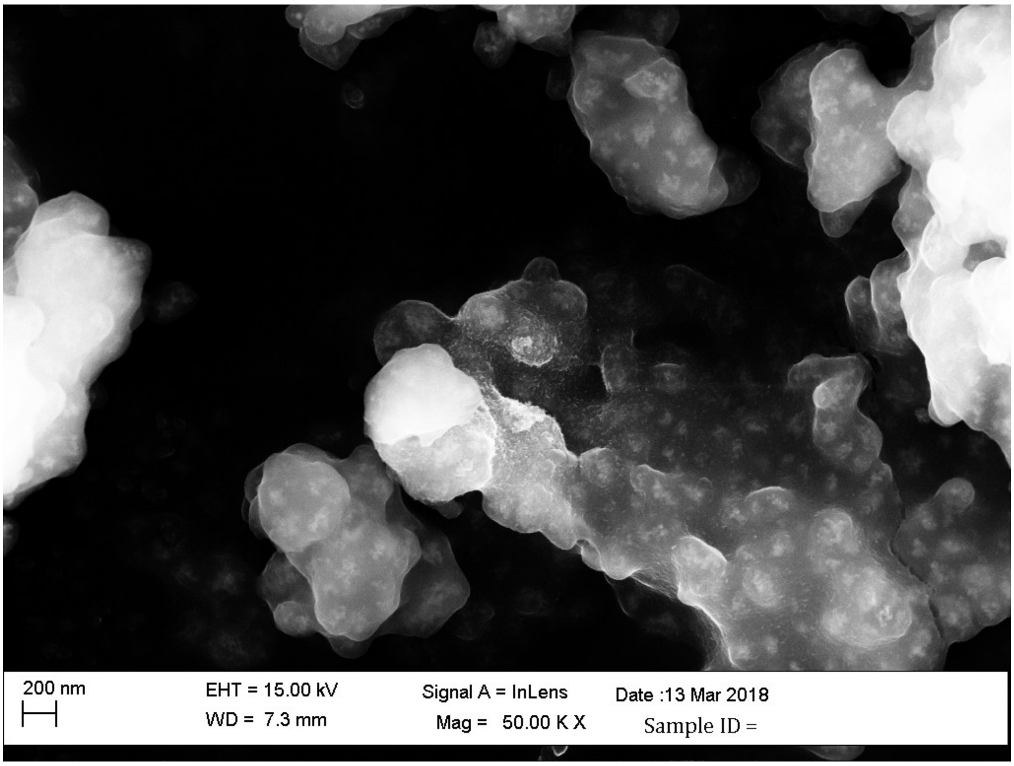

Figure S1. SEM image of material M0

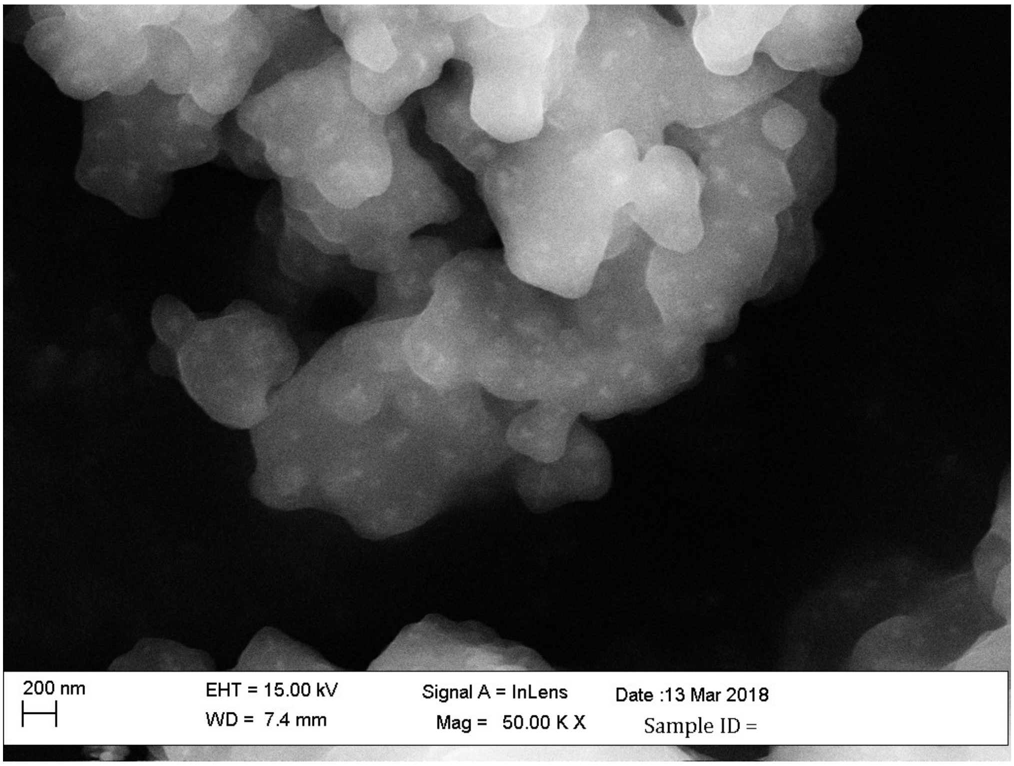

Figure S2. SEM image of material M1

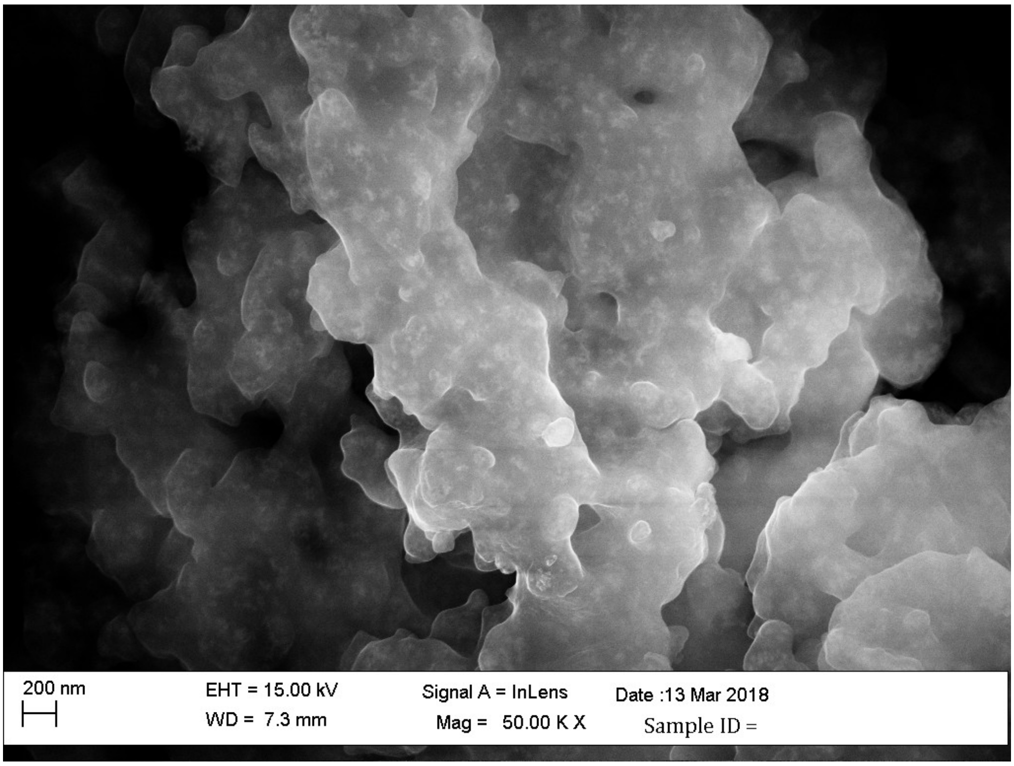

Figure S3. SEM image of material M2

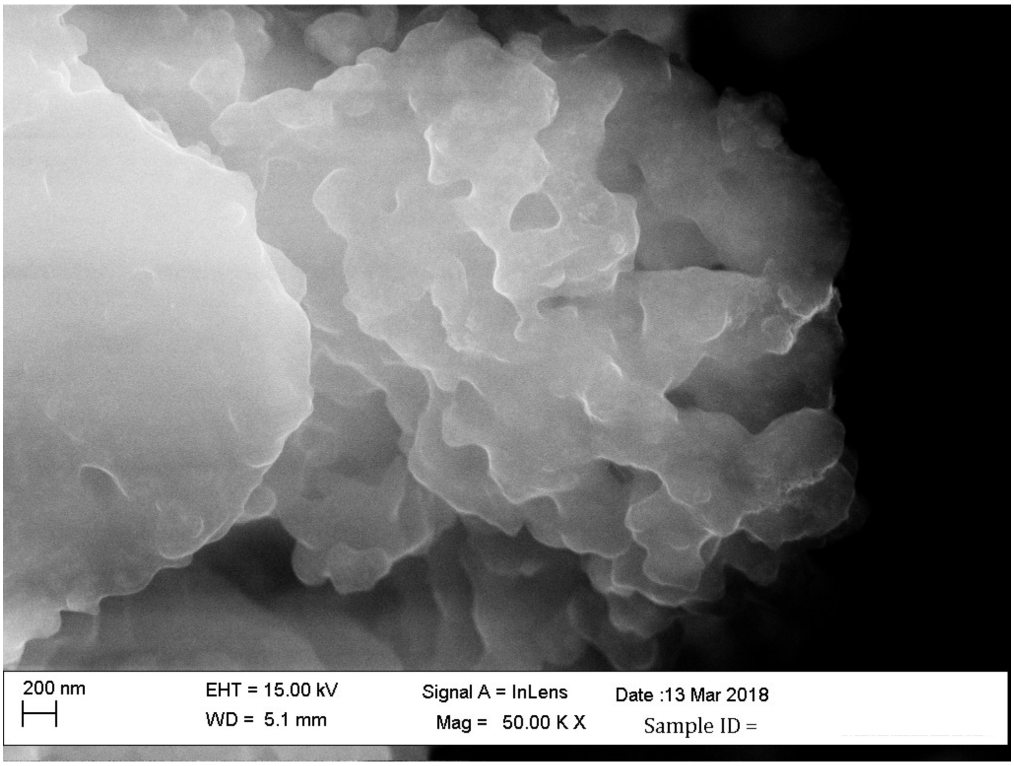

Figure S4. SEM image of material M3

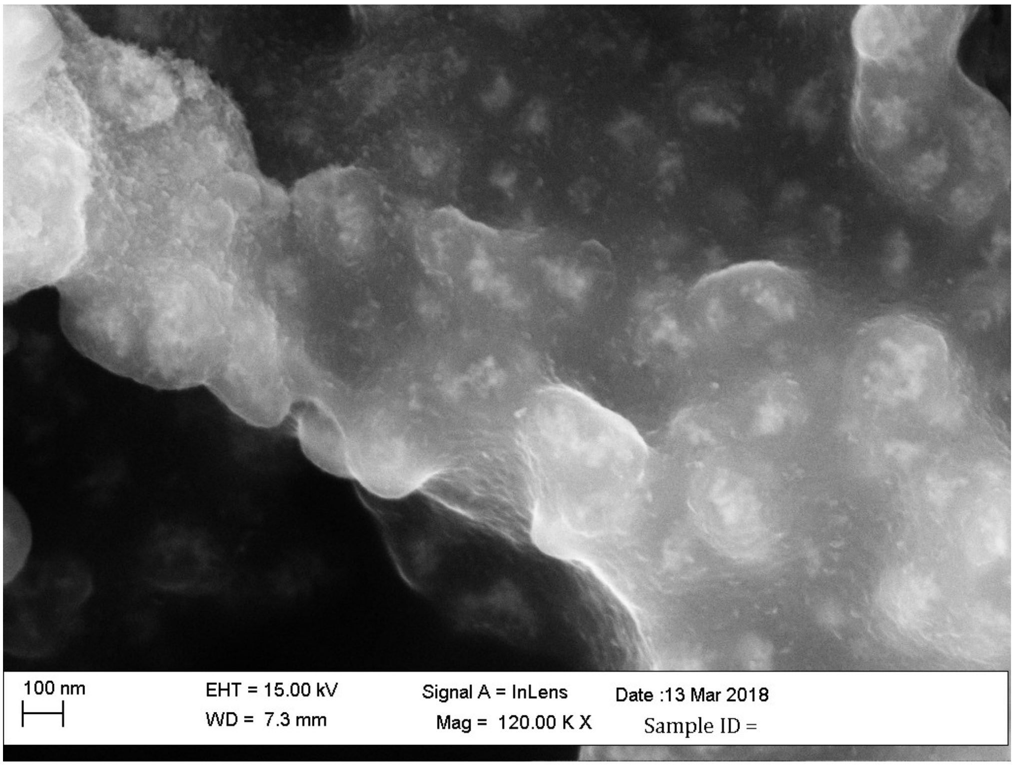

Figure S5. Detail of material M0 in which aggregates within the polymer shell are clearly visible and the size of which can be estimated to be around 100 nm.

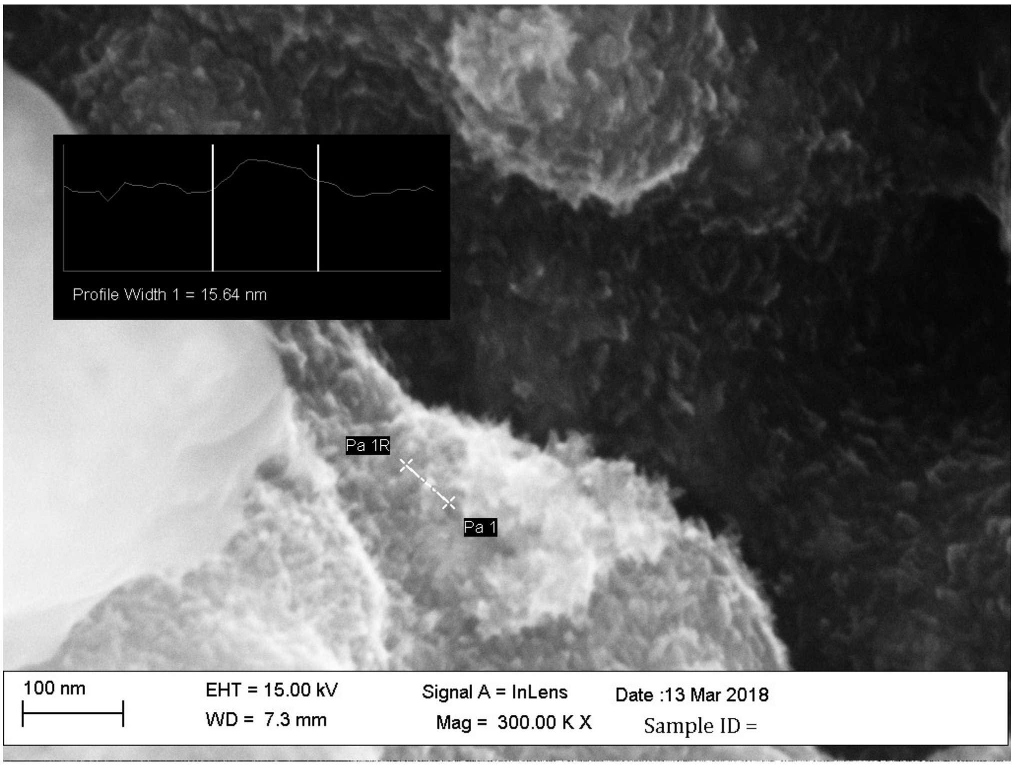

Figure S6. Detail of material M0 in which aggregates are magnified to show the nanoparticles which constitute them, which are around 15 nm.

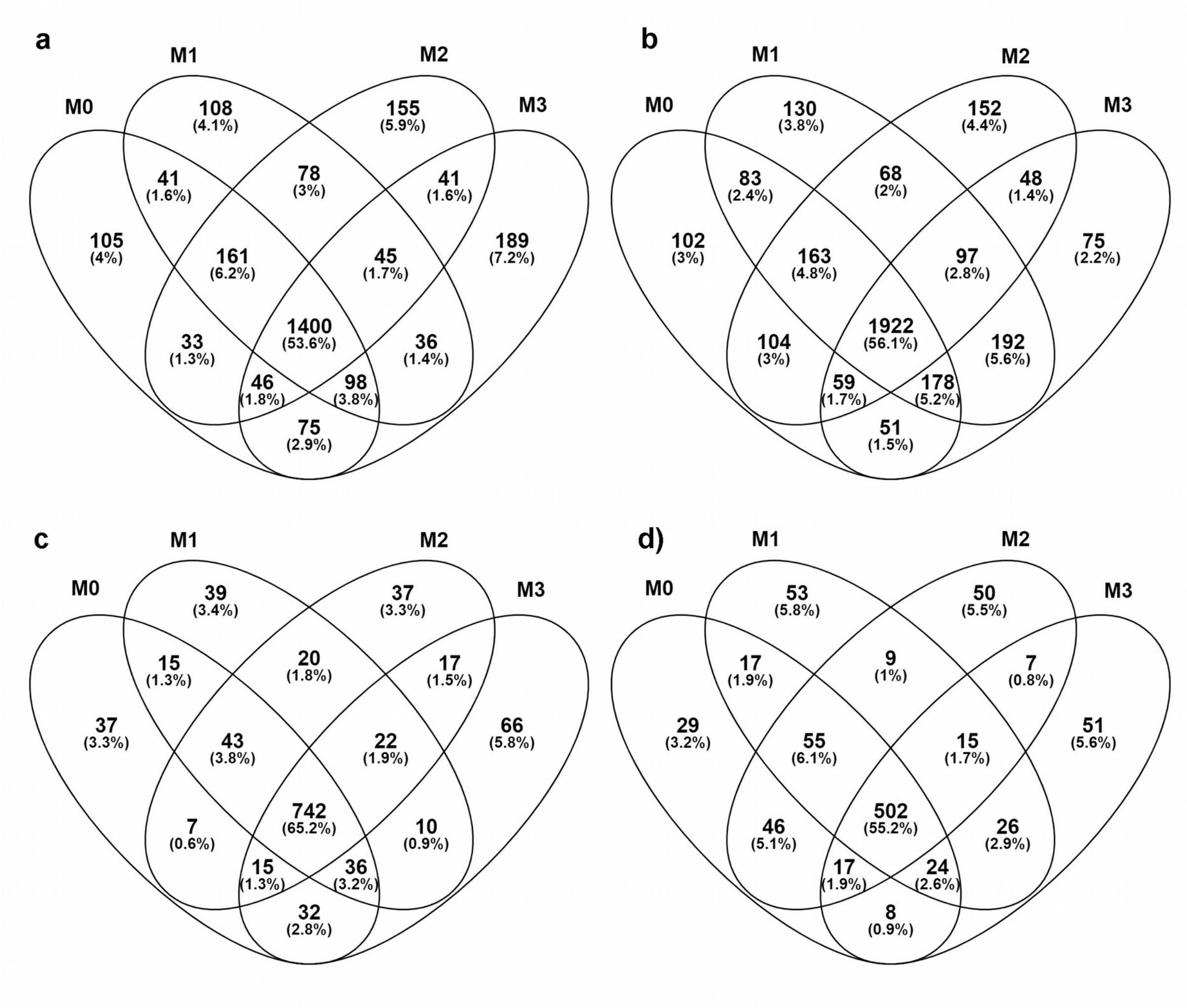

Figure S7. Venn diagrams for the distribution of the identified phosphopeptides (a), co-enriched non-phosphorylated peptides (b), phosphoproteins (c) and non-phosphorylated proteins (d) across the four tested materials.

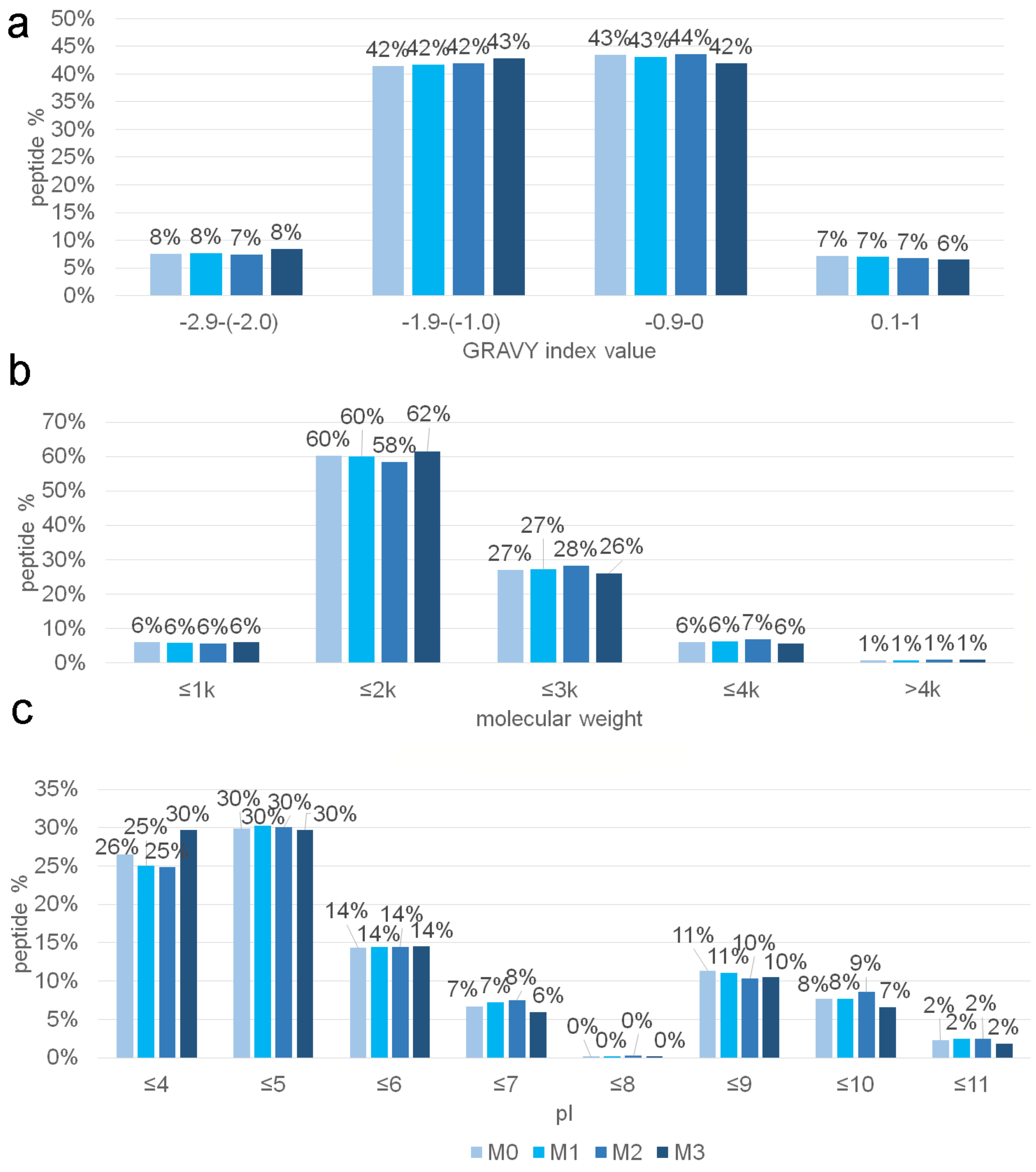

Figure S8. Distribution of GRAVY index value (a), molecular weight (b) and pI (c) of the identified phosphopeptides.
